# Kiwira Virus, a Newfound Hantavirus Discovered in Free-tailed Bats (Molossidae) in East and Central Africa

**DOI:** 10.3390/v14112368

**Published:** 2022-10-27

**Authors:** Sabrina Weiss, Lwitiho E. Sudi, Ariane Düx, Chacha D. Mangu, Nyanda Elias Ntinginya, Gabriel M. Shirima, Sophie Köndgen, Grit Schubert, Peter T. Witkowski, Jean-Jacques Muyembe, Steve Ahuka, Boris Klempa, Fabian H. Leendertz, Detlev H. Krüger

**Affiliations:** 1 Institute of Virology, Charité—University Medicine Berlin, Corporate Member of Free University Berlin, Humboldt-University Berlin, 10117 Berlin, Germany; 2 Robert Koch Institute, 13353 Berlin, Germany; 3 NIMR—Mbeya Medical Research Center, Mbeya P.O. Box 2410, Tanzania; 4 Helmholtz Institute for One Health, 17489 Greifswald, Germany; 5 Nelson Mandela African Institution for Science and Technology, Arusha Arusha P.O. Box 447, Tanzania; 6 Institut National de Recherche Biomédical, Kinshasa BP 1197, Democratic Republic of the Congo; 7 Biomedical Research Center of the Slovak Academy of Sciences, Institute of Virology, Dúbravská Cesta 9, 845 05 Bratislava, Slovakia; 8 Department of Microbiology and Virology, Faculty of Natural Sciences, Comenius University in Bratislava, 814 99 Bratislava, Slovakia

**Keywords:** hantavirus, *Mobatvirus*, *Hantaviridae*, *Mops condylurus*, bat, phylogeny

## Abstract

A novel hantavirus, named Kiwira virus, was molecularly detected in six Angolan free-tailed bats (*Mops condylurus*, family Molossidae) captured in Tanzania and in one free-tailed bat in the Democratic Republic of Congo. Hantavirus RNA was found in different organs, with the highest loads in the spleen. Nucleotide sequences of large parts of the genomic S and L segments were determined by in-solution hybridisation capture and high throughput sequencing. Phylogenetic analyses placed Kiwira virus into the genus *Mobatvirus* of the family *Hantaviridae,* with the bat-infecting Quezon virus and Robina virus as closest relatives. The detection of several infected individuals in two African countries, including animals with systemic hantavirus infection, provides evidence of active replication and a stable circulation of Kiwira virus in *M. condylurus* bats and points to this species as a natural host. Since the *M. condylurus* home range covers large regions of Sub-Saharan Africa and the species is known to roost inside and around human dwellings, a potential spillover of the Kiwira virus to humans must be considered.

## 1. Introduction

Hantaviruses carry tri-segmented, negative-sense RNA genomes and form a unique virus family, *Hantaviridae*. The small (S) genomic segment encodes the viral nucleocapsid protein (N), the medium (M) segment the envelope glycoproteins, and the large (L) segment the viral RNA-dependent RNA polymerase [1]. The viruses are hosted by small mammals and some of them can accidentally infect humans to cause kidney and pulmonary diseases of different severities [2].

Whereas rodent-borne hantaviruses from Europe, Asia, and the Americas have been described for many decades, molecular evidence for the first indigenous rodent-borne hantavirus on the African continent, the Sangassou virus, was reported as late as 2006 [3]. Soon thereafter, molecular detection of a shrew-borne hantavirus, Tanganya virus, was reported in West Africa, too [4]. As late as 2012, the first hantaviruses were described in bats from West Africa; Magboi virus from Sierra Leone [5] and Mouyassué virus from Côte d’Ivoire [6].

Today, it is known that bats, moles, shrews, and most importantly rodents, are natural hosts of mammalian hantaviruses. All known human pathogenic hantaviruses are rodent-borne and belong to the genus *Orthohantavirus*; they can cause febrile illnesses with renal and/or cardiopulmonary impairment and even organ failure [2]. While there is serological evidence of human hantavirus infections in Guinea, Côte d’Ivoire, Democratic Republic of the Congo (DRC), and Gabon, no hantavirus genetic material has yet been amplified from any patient’s specimen from Africa [7,8,9].

Ten different bat-borne hantaviruses have been described in 14 bat species in Asia, Europe, and Africa [10]. Often, only short sequences have been detected and/or the respective specimens originated only from single or very few animals—therefore, it remained unclear whether they were natural or spillover hosts. So far, none of these viruses has been isolated in cell culture and it is largely uncertain whether they can infect and cause disease in humans. Taxonomically, bat-borne hantaviruses are assigned to the genera *Loanvirus* and *Mobatvirus* of the *Hantaviridae* family; they are genetically distinct from orthohantaviruses [11]. Here, we report the discovery of Kiwira virus, a novel hantavirus infecting Angolan free-tailed bats (*Mops condylurus*), a species previously not known to harbour hantaviruses, and provide details on the tissue distribution of viral RNA and phylogenetic placement of the virus.

## 2. Materials and Methods

Bats were captured using mist nets in the Salonga National Park, in the western part of the Democratic Republic of Congo (DRC), in 2013 and near the Kiwira river, Mbeya region, South-West Tanzania (TZ), in 2017. Animals were sacrificed by anaesthesia with ketamine/xylazine followed by exsanguination through cardiac puncture. Weight, forearm length, sex, and age were recorded, and pictures of each individual were taken. Tissue specimens were collected following strict biosafety measures and immediately stored in liquid nitrogen. Samples were then shipped on dry ice and subsequently stored at −80°C until further processing. Ethical approval has been obtained from the local authorities and, in summary, by the Charité Ethics Committee (permission no. EA1/025/09).

RNA was extracted from frozen organ samples using the RNeasy Mini Kit (Qiagen, Hilden, Germany) and reverse-transcribed using M-MLV (Thermo Fisher Scientific, Waltham, MA, USA) and random hexamer primers. The resulting cDNA was used in a PAN-Hanta-PCR amplifying a 347 nucleotide (nt) long region of the genomic L segment [3] to screen for hantaviruses, and amplicons were Sanger sequenced on both strands. Based on the resulting sequences, a qPCR was designed to semi-quantify virus load in all available organ samples of animals from TZ that were positive in the PAN-Hanta-PCR (Kiw-L_fwd: CAgCAgCTCTTCACAATggT; Kiw-L_rev: TCCTCCTTCAgCTCCATggA; Kiw-L_TM: 6FAM-ACTgAATTCTTTCTgTCCAgAAgg-CT-BBQ).

To generate more sequence information, two tissue samples with high virus loads were selected and used for in-solution hybridisation capture followed by subsequent next-generation sequencing. Therefore, RNA was extracted with an RNeasy mini kit (Qiagen) and treated with TURBO DNAse (Ambion, Austin, TX, USA, Life Technologies, Carlsbad, CA, USA). First-strand cDNA synthesis was performed using the Superscript IV reverse transcriptase kit (Thermo Fisher Scientific) and FR26RV-N primer (Djikeng et al., 2008). Double-stranded (ds) cDNA was generated using the NebNext mRNA 2nd Strand Synthesis kit (New England Biolabs, Ipswich, MA, USA) and was then purified with Ampure XP beads (Agencourt). Sequence-independent single primer amplification (SISPA) was carried out with the Advantage 2 PCR kit (Takara, Shiga, Japan) and primer FR20RV (Djikeng et al., 2008). After a final purification step with Ampure XP beads (Agencourt), DNA concentrations were measured using a Qubit dsDNA High Sensitivity kit (Life Technologies). Samples were subsequently fragmented using a Covaris S220 Focused-ultrasonicator^®^ (Intensity = 4, Duty cycle = 10%, Cycles per burst = 200, Treatment time = 55 s, Temperature = 7 °C). Libraries were prepared using the NebNext Ultra II library kit (New England Biolabs) following the standard protocol, with dual indices. RNA baits (MYbaits, Arbor Biosciences) used for in-solution capture were based on publicly available hantavirus genomic sequences as well as unpublished sequences generated in our laboratory. Enrichment of hantavirus libraries was done using two rounds of hybridisation at 65 °C for 48 h, following the Mybaits Sequence Enrichment for Targeted Sequencing protocol (version 4.0). The product was purified with Ampure XP beads (Agencourt) and used as input for the Illumina MiSeq sequencer using the MiSeq Reagent Kit v2 (Illumina) with 2 × 300 cycles.

Sequences were trimmed, deduplicated, and mapped against various Mobatvirus genomes using Geneious Prime v10. Dakrong virus (MG663534-36), Laibin virus (NC_038513-15), Mouyassue virus (JQ287716), Nova virus (NC_034464, NC_034465, NC_034470), Quezon virus (NC_034393, NC_034400, NC_034401), and Xuan Son virus (KY662273-5) were used as reference genomes. Consensus sequences were generated with bases matching at least 60% of the total adjusted chromatogram quality and minimum 10-fold coverage. All consensus sequences of one sample and segment were then assembled, visually inspected, and cumulated to generate a single consensus sequence for each sample and segment. These were used as a reference to re-map all reads using bwa-mem 0.7.15-r1140 [12]. Final consensus sequences were generated using the abovementioned criteria.

For phylogenetic analyses, sequences were aligned at the amino acid level and alignments were optimised using Gblocks [13] as implemented in SeaView [14]. Maximum likelihood phylogenetic trees were calculated using PhyML3.0 [15] based on the model of nucleotide or amino acid evolution (SMS v1.8.4) as determined by Smart Model Test [16]. For preliminary taxonomic classification, pairwise evolutionary distances (PED) were calculated using a Whelan and Goldman (WAG) amino acid substitution model [17] and maximum likelihood approach implemented in TREE-PUZZLE [18].

## 3. Results

Using the PAN-Hanta-PCR, we found hantavirus sequences in 6/334 lung samples of bats that were captured in TZ and in 1/49 animals that were captured in the DRC. The hantavirus positive animals from TZ were identified as *Mops condylurus* by morphological characterisation and confirmed by cytB analysis. The bat species *Mops condylurus* (Angolan free-tailed bat) is a member of the family Molossidae (free-tailed bats) [19]. Five animals from TZ were male and one was female. The positive individual from DRC was male. We could not unambiguously determine the species either in the field or retrospectively. Because of a shortage of material, no cytB analysis was possible for this individual; however, it was, based on morphological data and photographs, clearly classified as a member of the Molossidae family but not *M. condylurus*. The negative animals were identified to belong to the families Pteropodidae (n = 226), Molossidae (n = 89), Vespertilionidae (n = 39, Rhinolophidae (n = 3), and Hipposideridae (n = 1), or remained unidentified (n = 18).

For the six positive animals from Tanzania, a detailed investigation of their lung, liver, kidney, spleen, and intestine tissues was conducted. One animal from Tanzania (TZ117) tested positive in the lung only, whereas, for the other individuals, virus RNA was found by qualitative PAN-Hanta-PCR in all investigated organs (Table 1, yellow shade). If sufficient tissue material was available, we performed Kiwira virus-specific quantitative PCR to assess the virus concentration in the different tissues (symbols + to +++ in Table 1). In animals TZ154 and TZ157, the highest relative virus loads were detected in spleen samples (Table 1). For the positive animal from DRC (DRC348), only lung tissue was tested by the PAN-hanta PCR. Moreover, tissues from the positive animals from Tanzania were also used for unsuccessful isolation attempts on Vero E6 cells (data not shown).

**Figure 1:**
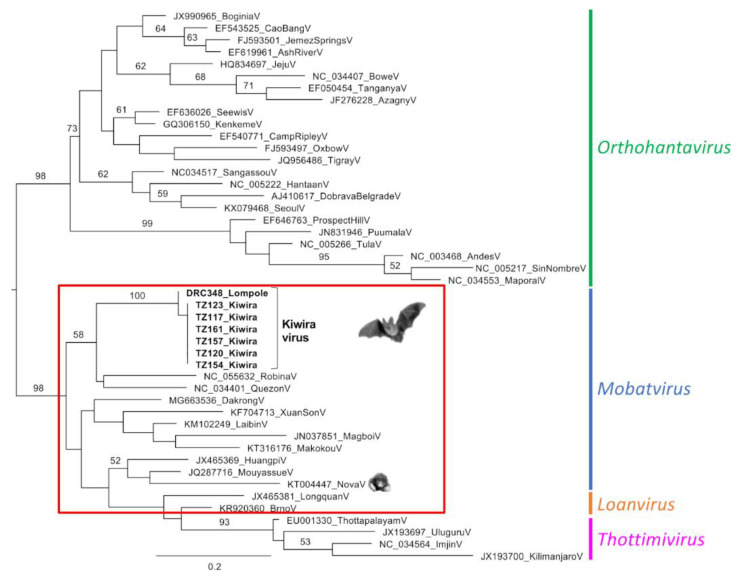
Maximum likelihood phylogenetic tree based on 142 amino acids (aa) of the L segment. Trees were inferred using the LG + G + I model with 1000 bootstraps. Bootstrap values are given in percent and only when above 50%. Viruses from this study are given in bold. Scalebar indicates aa substitutions per site.

Pairwise comparison of the deduced amino acid sequences of the Pan-Hanta-PCR product revealed 98.6% identity between viruses from TZ and DRC. To other hantaviruses, the highest pairwise identities were observed for the bat-borne Quezon virus (81.4–82.9%) and Robina virus (80.0–81.4%) (Table 2). Consistently, phylogenetic analyses of the same fragment placed the novel virus strains within the genus *Mobatvirus* and suggested Quezon and Robina viruses as the most closely related known hantaviruses (Figure 1).

We used spleen samples of individuals TZ154 and TZ157 for in-solution capture and high throughput sequencing and were able to obtain 4566 and 4875 nt of the L segment and 317 and 329 nt of the S segment, respectively. Despite the effort, no additional sequence information could be obtained for the sample from DRC. The highest pairwise amino acid identities of the longer sequences of TZ154 and TZ157 were again observed for Quezon virus (87.4 and 85.4% for the N protein and 82.9 and 80.4%, for the L protein sequences, respectively) and even slightly higher for Robina virus (88.3 and 86.4% for the N protein and 83.7 and 81.6%, for the L protein sequences, respectively) (Table 3). Moreover, we calculated PED values by using the WAG evolutionary model. The lowest PED values of 0.13 and 0.22 for the N and L protein sequences, respectively, were obtained for the Robina virus.

**Figure 2:**
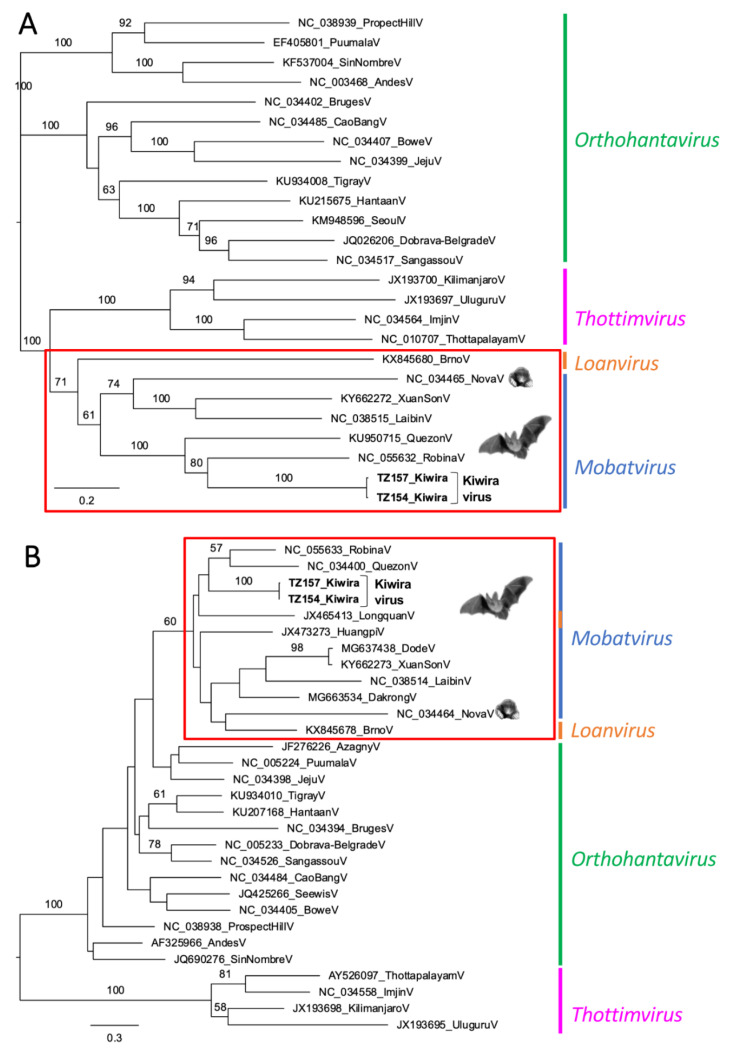
Maximum likelihood (ML) phylogenetic trees of partial L and S segments: (**A**) ML phylogeny based on a 4826 nt alignment of the L segment, calculated using the GTR +I +G model; (**B**) ML phylogeny based on 308 nt alignment of the S segment, calculated using the HKY85 +I +G model. Scalebars indicate nucleotide (nt) substitutions per site. Bootstrap values are based on 1000 replicates and shown when above 50%. Viruses from this study are given in bold.

Phylogenetic analyses of the longer fragments confirmed the phylogenetic placement of the novel virus sequences from TZ, called “Kiwira”, within the *Mobatvirus* genus. In the L segment analysis, Kiwira sequences directly clustered with the Robina virus and formed a well-supported monophyletic group with both Robina and Quezon viruses (Figure 2A). The same monophyletic group was also formed in the S segment analysis, although in this case, Robina and Quezon viruses clustered together and formed a sister group to the Kiwira sequences (Figure 2B). It should be noted that as a consequence of the too-short and incomplete S segment dataset, the position of other bat-borne viruses is not statistically supported and, in some cases (such as the Longquan virus), contradicts its taxonomical classification.

## 4. Discussion

We describe the detection of novel hantavirus sequences in bats from Sub-Saharan Africa. The virus sequences from Tanzania studied in detail were found in *M. condylurus*, a bat species not previously described to harbour hantaviruses. When the short virus L segment sequences from PCR screening were compared, a very high sequence similarity and molecular phylogenetic relatedness (Table 2, Figure 1) were observed between the strains from Tanzania (Kiwira TZ117, TZ120, TZ123, TZ154, TZ157, TZ161) and the strain from DRC (Lompole DRC348) found in another molossid species. Phylogenetic analysis placed the virus sequences in the genus *Mobatvirus*. For Kiwira TZ154 and TZ157, the analysis of larger fragments (L segment > 4500 nt; S segment > 300 nt) confirmed their placement in the genus *Mobatvirus* (Table 3, Figure 2). The data suggest that the sequences described here are representative of a novel hantavirus within this genus. We tentatively named it the Kiwira virus, based on the geographic location where animals in Tanzania were captured.

The current classification of the International Committee on Taxonomy of Viruses (ICTV) is based on PED values calculated by using the WAG amino acid substitution matrix from concatenated complete nucleocapsid (N) and glycoprotein precursor (GPC) amino acid sequences. A species is defined by a PED value greater than 0.1 [11]. Despite the efforts, we could not determine the complete N and GPC sequences and, therefore, currently cannot determine the required PED values. Nevertheless, we calculated the PED values for the obtained partial N and L protein amino acid sequences. The lowest PED values of 0.13 (N) and 0.22 (L) were observed for the Robina virus and indicate that the newly identified virus most likely represents a new species within the genus *Mobatvirus*. Moreover, the two recognised species, *Quezon mobatvirus* [20] and *Robina orthohantavirus* showed an under-the-cutoff PED value of only 0.07 for the analysed partial N protein sequence, indicating that this sequence fragment, in fact, underestimates the PED value calculated from the complete concatenated sequences.

It should be noted that the Robina virus has been classified as a new hantavirus species *Robina orthohantavirus,* within the latest taxonomic update [21]. However, in phylogenetic analyses, this virus unambiguously clusters with other bat-borne hantaviruses belonging to the genus *Mobatvirus*. Its current classification into the genus *Orthohantavirus*, therefore, seems to be incorrect and should be revised. Robina virus is the first and currently the only hantavirus recognised in Australia. It was discovered in a black flying fox (*Pteropus alecto*) sampled near Robina, Queensland, Australia, in 2017. However, any further details are yet to be published, and the species classification was solely based on the GenBank entries (NC_055632-4).

The hosts of Kiwira virus and its closest relatives, Robina virus and Quezon virus, are only distantly related. While *M. condylurus* belongs to the insectivorous bat family *Molossidae* (free-tailed bats), the putative hosts of Robina virus, *Pteropus alecto*, and Quezon virus, *Rosettus amplexicaudatus*, belong to the frugivorous *Pteropodidae* family (flying foxes), all in the order Chiroptera (bats) [19]. Beyond these two closest relatives, Kiwira virus nests within a bat-dominated clade whose weakly supported deep structure is compatible with a variety of evolutionary scenarios involving cross-species transmission events. If cross-species transmission occurred, they did so in the very distant past, given the observed extensive genetic divergence. Nevertheless, the detection of three closely related hantaviruses in taxonomically as well as ecologically diverse bats collected across three continents is quite surprising and further illustrates that the known diversity of bat-borne hantaviruses is vastly incomplete [22].

Kiwira virus represents the fourth bat-borne hantavirus identified in Africa, after the Magboi virus in Sierra Leone, the Makokou virus in Gabon, and the Mouyassué virus in Côte d’Ivoire and Ethiopia [5,6,22,23]. Kiwira virus infection of *M. condylurus* individuals was identified by virus RNA detection in their lungs. Aerosolised urine, among other excreta/secreta, is suggested as one of the main routes of hantavirus transmission among rodents as well as between rodents and humans [24,25]. Five of the six individuals for which a full set of organ samples was available showed systemic infection with the Kiwira virus, including involvement of the kidneys and intestines, which is suggestive of virus excretion through urine and faeces.

**Figure 3:**
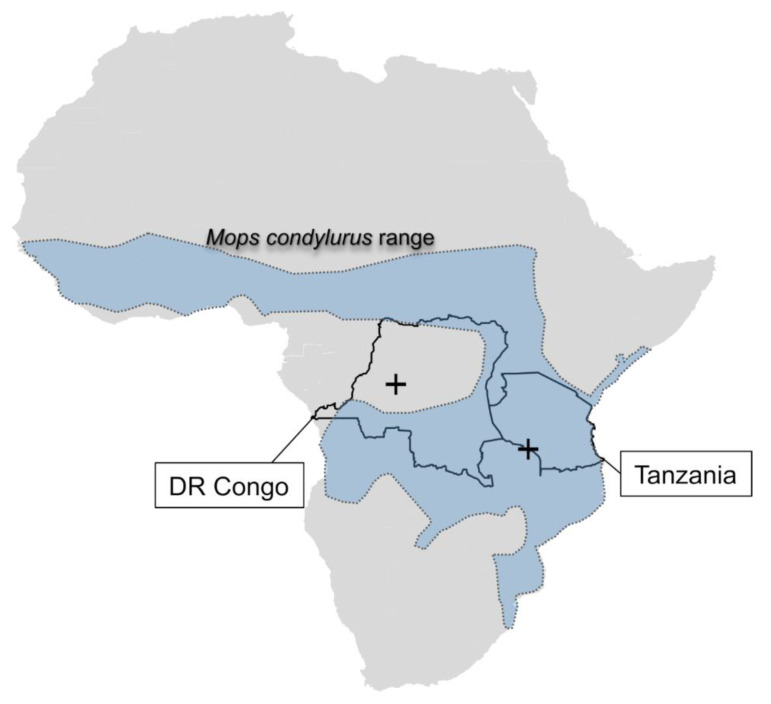
Finding places of bat-borne Kiwira hantavirus in Africa. Detection sites in Mbeya Region (Tanzania) and Salonga National Park (DRC) are marked by crosses. The approximate geographical range of the host species *Mops condylurus* is marked by a dashed line and blue shading (adapted from www.iucnredlist.org, accessed on 1 September 2022).

*M. condylurus* is present in wide regions of Sub-Saharan Africa [26]. The species is also a host of the recently discovered ebolavirus, Bombali virus, found in bats in West Africa but also 750 km away in East Africa [27,28]. *M. condylurus* animals are known to commonly roost in buildings and hollow trees; their distribution range spreads across the tropical and savannah regions from West to East Africa (Figure 3). It is noteworthy that animals in this study were captured in close proximity to human settlements.

There is no evidence yet that bat-borne hantaviruses can cause disease in humans, but it should be noted that hantavirus disease often manifests as a febrile illness with non-specific symptoms. These clinical symptoms are quite common for infections by several pathogens, particularly in Africa, and might be easily overlooked. Despite some efforts, robust and specific serological assays to account for infections caused by the increasing diversity of bat, shrew, and mole-associated hantaviruses are still lacking [9]. Development of such tools could help to shed light on the impact which bat-borne hantaviruses might have on human health. In a general seroepidemiological study in DRC, 7/295 unselected individuals were found to be hantavirus-antibody positive (without specifying the virus type) based on a sophisticated algorithm of screening and confirmatory assays [8]. Moreover, also shrew-borne hantaviruses with unknown pathogenic potential have been identified in Tanzania [29].

There are few reports on the ecology of bat-borne hantaviruses in Asia. Two of those, Longquan and Xuan son viruses, have even been described in more than one bat species [30,31,32]. In contrast, reports of African bat-borne hantaviruses were limited to single findings, and systemic infection has only been shown for the Makokou virus [22]. The detection of several individuals with systemic hantavirus infection in our study argues against the possibility of an accidental spillover and provides evidence of active replication and a stable circulation of the Kiwira virus in *M. condylurus* bats. Follow-up studies investigating bats of this species in the same region will not only give more insight into the distribution and biological properties of the Kiwira virus but also help to improve our overall understanding of the ecology of bat-borne hantaviruses in Africa.

## Figures and Tables

**Table 1:** Summary of hantavirus test results ^1^ of samples from Tanzania and DRC.

Sample ID	Sex	Location	GPS	Lung	Liver	Kidney	Spleen	Intestine
TZ117	male	Kajunjumele	−9.609889, 33.913750	+	-	-	-	NA
TZ120	male	Kajunjumele	−9.609889, 33.913750		+	+		NA
TZ123	male	Kajunjumele	−9.609889, 33.913750			NA	NA	
TZ154	male	Kyela	−9.602364, 33.925929	+	+	+	++	+
TZ157	female	Kyela	−9.602364, 33.925929	++	+	+	+++	+
TZ161	male	Kyela	−9.602364, 33.925929					
DRC348	NA	Lompole	−2.5881744, 20.3696069		NA	NA	NA	NA

^1^ Yellow shade = PAN-hanta-PCR positive; Relative quantification of virus load by Kiwira-specific qPCR (if available): +++, Ct value 20–24; ++, Ct value 25–29; +, Ct value 30–35; -, PAN-hanta-PCR negative; NA = not available.

**Table 2:** Pairwise identities [%] between sequences from Tanzania and the Democratic Republic of Congo and their closest relatives, Quezon virus and Robina virus ^1^.

	NC_034401 Quezon Virus	NC_005632 Robina Virus	DRC348 Lompole
TZ117	82.1	80.0	98.6
TZ120	81.4	80.0	98.6
TZ123	82.1	80.7	98.6
TZ154	82.1	80.0	98.6
TZ157	82.1	80.7	98.6
TZ161	82.1	80.7	98.6
DRC348	82.9	81.4	

^1^ Analysis based on the amino-acid alignment of the Pan-Hanta PCR L segment (142 aa).

**Table 3:** Pairwise identities between sequences from Tanzania and their closest relatives, Quezon virus and Robina virus [%] ^1^.

Kiwira Virus Samples	Pairwise Identity [%] to Quezon Virus ^2^	Pairwise Identity [%] to Robina Virus ^3^	Number of Mapped Reads
TZ154S	87.4	88.3	39,234
TZ157S	85.4	86.4	2480
TZ154L	82.9	83.7	1,997,514
TZ157L	80.4	81.6	1,478,369

^1^ Analysis based on amino-acid (aa) alignments of the identified Kiwira virus S and L sequences (105 and 1,645 aa, respectively). The number of mapped reads relates to the Quezon virus. ^2^ S segment: NC_034400; L segment: KU950715. ^3^ S segment: NC_055633; L segment: NC_055632.

## Data Availability

All viral genomic nucleotide sequences obtained within this study have been submitted to the GenBank database under Accession Numbers OP313834-8 and OP122966-9.
